# Tempol Attenuates Neuropathic Pain by Inhibiting Nitric Oxide Production

**DOI:** 10.1155/2019/8253850

**Published:** 2019-05-15

**Authors:** Donglin Jia, Huan Wang, Bin Han, Liping Zhang, Jianrong Guo

**Affiliations:** ^1^Department of Pain Medicine, Peking University Third Hospital, Beijing 100191, China; ^2^Department of Anesthesiology, Gongli Hospital, The Second Military Medical University, Pudong New District, Shanghai 200135, China; ^3^Department of Anesthesiology, Peking University Third Hospital, Beijing 100191, China

## Abstract

**Background:**

Neuropathic pain not only affects individual life quality but also increases economic burden for the society. Treatment to alleviate neuropathic pain is required.

**Methodology:**

Fifty rats were randomly assigned into sham, spinal nerve ligation, and three treatment groups with different doses of Tempol (100, 200, and 300 mg/kg, respectively), with 10 rats in each group. A neuropathic pain model was created with spinal nerve L5 and L6 ligation. Mechanical allodynia and thermal hyperalgesia were tested preoperatively (day 0) and postoperatively (days 1, 3, 5, and 7). Spinal cord levels of nitric oxide, as well as activities of nitric oxide synthase and acetylcholinesterase, were tested in postoperative day 7.

**Results:**

Compared with rats in the spinal nerve ligation group, rats in Tempol treatment groups had decreased responses to mechanical pain and cold plate stimulations. A high dose of Tempol produced more attenuating effects. The level of nitric oxide and activity of nitric oxide synthase were also decreased with Tempol treatments, whereas no significant changes were observed in the activity of acetylcholinesterase.

**Conclusions:**

Tempol attenuated an experimental rat model with neuropathic pain by inhibiting nitric oxide production.

## 1. Introduction

Neuropathic pain is caused by primary disorders in the peripheral or central nervous system [1]. It is a type of chronic pain which not only affects individual life quality but also increases economic burden for the society [2]. Treatments, such as antidepressants, anticonvulsants, or opioids, were recommended for neuropathic pain, but many patients could not get a satisfactory response or experience unwanted adverse effects [3, 4]. Therefore, the development of new therapeutic strategies for neuropathic pain is required.

Many factors have been proved to contribute to the development of neuropathic pain. Reactive oxygen species and some neurotransmitters, such as acetylcholine, are involved in the pathogenesis of neuropathic pain [5–8]. Administration of antioxidants could improve the pain score and quality of life in patients with neuropathic pain [9]. Tempol (4-hydroxy-2,2,6,6-tetramethylpiperidine N-oxyl) is a newly identified antioxidant. Studies have shown that Tempol could increase sensitivity to insulin treatment and slow the hypertension development in experimental rat models [10]. However, there were limited studies on the effects of Tempol in the treatment for neuropathic pain [11].

In the current study, we reported the effects of Tempol on an experimental rat model with neuropathic pain. We studied rat behavior changes, as well as the levels of nitric oxide, and activities of nitric oxide synthase and acetylcholine. Our results showed that Tempol could attenuate rat neuropathic pain, probably from inhibition of nitric oxide production.

## 2. Materials and Methods

### 2.1. Animals

Fifty adult Sprague-Dawley rats (male, weighing 180–200 g) were obtained from the Animal Care Center at the Department of Medicine of Beijing University. They were fed standard rat chow and water *ad libitum* and were maintained individually in plastic boxes on a 12-hour light-dark cycle with room temperature. The study protocol was approved by the Animal Ethics Committee of Beijing University, and all procedures were conducted in accordance with the National Institutes of Health Guidelines for the Care and Use of Laboratory Animals and the Animal Welfare Act.

### 2.2. Study Protocol

Rats were randomly assigned into 5 groups, namely, sham group, spinal nerve ligation (SNL) group, low Tempol group (100 mg/kg), middle Tempol group (200 mg/kg), and high Tempol group (300 mg/kg), with each group containing 10 rats. The rat neuropathic pain model was created by spinal nerve ligation as described previously [12]. Briefly, rats were placed in the prone position under chloral hydrate (300 mg/kg, intraperitoneal injection) anesthesia. A skin incision was made at the midline of the back. After separation of the left paraspinal muscles and partial removal of the L6 transverse process, L4, L5, and L6 nerve roots were exposed. Except rats in the sham group, rats in all other groups received L5 and L6 nerve ligation by a 6-0 silk thread. All rats received one million units of penicillin intramuscular injection postoperatively to prevent infection. Rats in Tempol groups also received 100, 200, and 300 mg/kg Tempol injection peritoneally for low-, middle-, and high-dose groups, respectively. Mechanical allodynia and cold pain thresholds were measured preoperatively (day 0) and on days 1, 3, 5, and 7 postoperatively. At the postoperational day 7, rats were killed and the levels of spinal fluid nitric oxide, and activities of nitric oxide synthase and acetylcholinesterase, were measured.

### 2.3. Measurement of Mechanical Allodynia

Mechanical allodynia was quantified by 50% paw withdrawal in responses to mechanical stimulations from von Frey filaments using the up-down method [13]. Briefly, rats were habituated for 15 min in a plexiglass chamber with a metal mesh floor. The von Frey filament was applied for 4 seconds to the midplantar surface of the rat left hind paw. Positive response was recorded when paw lifting or licking was observed. Paw withdrawals induced by body movements were not counted as positive reactions. A series of 8 von Frey filaments with forces 1.0, 1.4, 2.0, 4.0, 6.0, 8.0, 10.0, and 15.0 g were used. First, a filament with 2.0 g force was applied. Then, a filament with the next weaker force was tested if there was a positive response, whereas a filament with the next stronger force was tested if there was a negative response. These tests were continued with progressively increasing or decreasing forces until there was a response different from the previous test. Every filament with different forces was tested 5 times with a 30-second interval. The number of positive responses for each filament tested was recorded. If the positive responses were no less than 3 times in these 5 stimulations, a positive rate for maximal force was calculated as the percentage of positive response. If the positive responses were less than 3 times, the positive rate for minimal force was calculated. Fifty percent paw withdrawal threshold was calculated as maximal force − [(maximal force − minimal force)/(positive rate for maximal force − positive rate for minimal force)] × (positive rate for maximal force − 50%). Fifty percent paw withdrawal threshold was recorded as 15.0 g or 1.0 g if more than 15 g or less than 1.0 g was required to induce a positive response.

### 2.4. Measurement of Thermal Hyperalgesia

Rat responses to cold stimulations were tested by placing rats on a cold plate with a temperature 5 ± 1°C. After rat exploratory activities ceased in 5 min, the number of paw withdrawal in 5 min was recorded by an inspector unknown to rat group assignment. Paw withdrawals due to body activities or movements were not recorded.

### 2.5. Measurements of the Level of Spinal Cord Nitric Oxide and Activities of Nitric Oxide Synthase and Acetylcholinesterase

At the postoperative day 7 after completing behavioral tests, rats were killed, and their spinal cords in the bulging lumbar segment were obtained. After washing the blood away with cold normal saline, spinal cord tissue homogenates were made by grinding spinal cord tissue in the homogenizers. The level of nitric oxide (NO) and activities of nitric oxide synthase (total (tNOS) and inducible (iNOS) forms) and acetylcholinesterase were tested according to the protocols provided by the manufacturer (Nanjing Jiancheng Bioengineering Institute, Nanjing, China).

### 2.6. Data Analyses

Study outcomes included 50% paw withdrawal threshold, number of paw lifting on the cold plate, level of spinal cord NO, and activities of NOS (tNOS and iNOS) and acetylcholinesterase. Results were presented as mean ± standard deviation. The one-way ANOVA test was used to compare the differences among different groups (SPSS, version 13.0). *P* < 0.05 was considered statistically significant.

## 3. Results

### 3.1. Mechanical Allodynia and Thermal Hyperalgesia

There were 10 rats in each study group, with a total of 50 rats. All rats completed the study.

**Figure 1:**
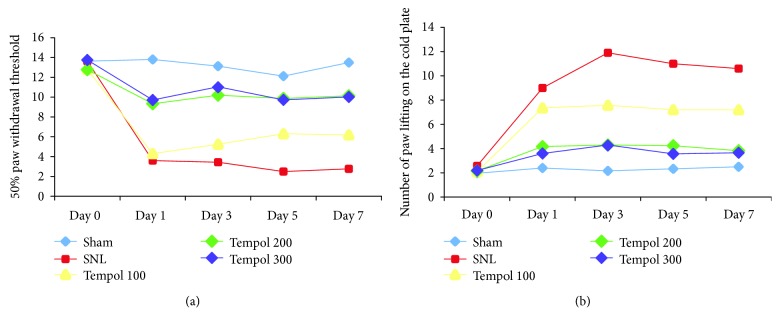
Effects of Tempol on 50% paw withdrawal threshold (a) and number of paw lifting on the cold plate (b) in different experimental groups (^∗^*P* < 0.05 compared with the sham group; ^#^*P* < 0.05 compared with the SNL group).

Compared with preoperative day 0, postoperative rats in the sham group showed no statistically significant changes in 50% paw withdrawal threshold and number of paw lifting on the cold plate, whereas rats in the SNL group had a statistically significant decreased 50% paw withdrawal threshold and increased number of paw lifting on the cold plate (Figure 1).

Compared with rats in the SNL group, rats in Tempol treatment groups had less significant decreases in 50% paw withdrawal threshold and increases in the number of paw lifting on the cold plate. These effects were observed on postoperative day 1 and persisted till the completion of the study (day 7). Compared with rats in the low-dose Tempol group (100 mg/kg), rats that received a high dose of Tempol (200 and 300 mg/kg) injections had more significant differences than the SNL group (Figure 1).

### 3.2. Levels of Nitric Oxide and Activities of Nitric Oxide Synthase and Acetylcholinesterase

**Figure 2:**
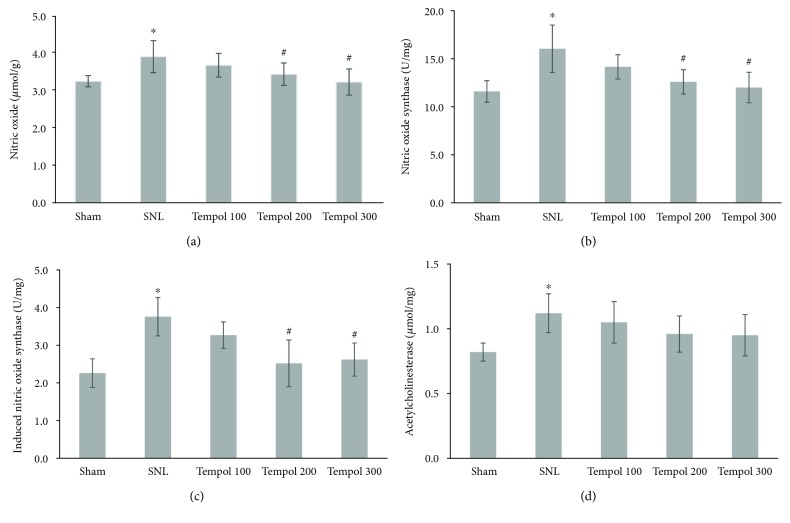
Spinal cord level of nitric oxide (a) and activities of total nitric oxide synthase (b), induced nitric oxide synthase (c), and acetylcholinesterase (d) in different experimental groups (^∗^*P* < 0.05 compared with the sham group; ^#^*P* < 0.05 compared with the SNL group).

Compared with the sham group, postoperative rats in the SNL group had an increased NO level and NOS and acetylcholinesterase activities. With increasing doses of Tempol, rats showed a statistically significant decreased NO level and NOS activity (Figure 2).

## 4. Discussion

In the current study, we showed that Tempol could alleviate mechanical allodynia and thermal hyperalgesia in an experimental rat model with neuropathic pain. Tempol also decreased the spinal cord NO level and NOS activity. NO is a free radical molecule, and its production is controlled by NOS. Free radicals are involved in the development of neuropathic pain. Our results demonstrated that Tempol could attenuate neuropathic pain via inhibition of nitric oxide production.

Neurogenic pain is due to disorders in the peripheral or central nervous system and is characterized by hyperalgesia, paresthesia, and spontaneous pain [1]. Recent studies have shown that oxygen free radicals were involved in the development and maintenance of neurogenic pain. Substances with antioxidant activity could alleviate oxygen free radical activities and potentially alleviate neurogenic pain. Tempol is a new antioxidant and has been shown to reduce oxidative stress, improve insulin sensitivity, and alleviate experimentally induced mechanical hyperalgesia. However, there were limited studies on the effects of Tempol on neurogenic pain [11].

In the current study, we have successfully established a neurogenic pain model with L5 and L6 spinal nerve root ligation. Rats with only spinal nerve root ligation but without any Tempol treatment had significantly decreased 50% paw withdrawal threshold and increased number of paw lifting on the cold plate. With Tempol treatment, rats showed a dose-dependent alleviation on these two measured outcomes, with higher doses having more protective effects against experimentally induced pain. This suggested that Tempol could attenuate neuropathic pain in these experiment rats.

In the current study, we further investigated a potential mechanism of Tempol on neuropathic pain. We selected to test the spinal cord NO level and NOS activity, since NO is a free radical molecule and was reported to be involved in neuropathic pain development [14]. NO synthesis is controlled by a rate-limiting enzyme NOS [15]. A calcium-independent oxide synthase (iNOS) is an important form of NOS and is involved in infection and inflammatory processes [16]. An increased level of iNOS could result in elevated production of NO, which further activates astrocytes. These activated astrocytes can produce a large amount of prostaglandin and result in increased neuron excitability to painful stimulations [17]. Our study results showed that rats in the SNL group had an increased level of NO and activities of TNOS and iNOS than rats in the sham group, which suggested that increased pain susceptibility could be due to increased production of NO. Tempol treatment could decrease NO production, with associated decreased susceptibilities to mechanical and thermal stimulations. These suggested that the pain attenuation effects of Tempol might be mediated through inhibiting NOS and, thus, NO productions.

Acetylcholine participates in the regulation of neuron activities. Previous studies reported that acetylcholine was involved in the neuropathic pain [7, 8]. In the current study, we found that the SNL group had increased acetylcholinesterase activity when compared with the sham group. Acetylcholinesterase is the primary enzyme for acetylcholine metabolism. Increased acetylcholinesterase activity in the SNL group suggested that the decreased acetylcholine level was involved in the neuropathic pain. This was consistent with previous study reports [18]. We did not observe significant differences in spinal cord acetylcholinesterase activities between SNL and Tempol groups, suggesting that effects of Tempol on neuropathic pain might not be mediated by acetylcholine.

In conclusion, Tempol could attenuate the experimental rat model with neuropathic pain. This effect might be mediated by inhibiting NO production. Tempol, as a new type of antioxidant, have an advantage of small molecular weight, stable properties, good cell permeability, and nontoxicity. Although Tempol has not been reported to be used to cure neuropathic pain in the clinical practice, animal experimental results have shown good prospects.

## Data Availability

The data used to support the findings of this study are available from the corresponding author upon request.
